# Barriers to Treg therapy in Europe: From production to regulation

**DOI:** 10.3389/fmed.2023.1090721

**Published:** 2023-01-19

**Authors:** Conor Hennessy, Milena Deptula, Joanna Hester, Fadi Issa

**Affiliations:** ^1^Transplantation Research and Immunology Group, Nuffield Department of Surgical Sciences, University of Oxford, Oxford, United Kingdom; ^2^Laboratory of Tissue Engineering and Regenerative Medicine, Division of Embryology, Medical University of Gdańsk, Gdańsk, Poland

**Keywords:** Treg, regulation, production, ATMP, cell therapy

## Abstract

There has been an increased interest in cell based therapies for a range of medical conditions in the last decade. This explosion in novel therapeutics research has led to the development of legislation specifically focused on cell and gene based therapies. In Europe, the European medicines agency (EMA) designates any medicines for human use which are based on genes, tissues, or cells as advanced therapy medicinal products or advanced therapy medicinal products (ATMPs). In this article we discuss the hurdles to widespread adoption of ATMPs in Europe, with a focus on regulatory T cells (Tregs). There are numerous barriers which must be overcome before mainstream adoption of Treg therapy becomes a reality. The source of the cells, whether to use autologous or allogenic cells, and the methods through which they are isolated and expanded, must all meet strict good manufacturing practice (GMP) standards to allow use of the products in humans. GMP compliance is costly, with the equipment and reagents providing a significant cost barrier and requiring specialized facilities and personnel. Conforming to the regulations set centrally by the EMA is difficult, and the different interpretations of the regulations across the various member states further complicates the regulatory approval process. The end products then require a complex and robust distribution network to ensure timely delivery of potentially life saving treatments to patients. In a European market whose logistics networks have been hammered by COVID and Brexit, ensuring rapid and reliable delivery systems is a more complex task than ever. In this article we will examine the impact of these barriers on the development and adoption of Tregs in Europe, and potential approaches which could facilitate more widespread use of Tregs, instead of its current concentration in a few very specialized centers.

## Introduction

Cell therapies have seen a surge in interest in the last decade as potential treatments for a range of challenging medical conditions. The European medicines agency (EMA) defines any medicines for human use which are based on genes, tissues or cells as “advanced therapy medicinal products (ATMPs),” a definition which encompasses any cell based therapies for human use. To date, over 500 trials using ATMPs have been performed, yet only 23 ATMPs currently have marketing authorization (MA) under the EMA. The disparity between the number of trials and the number of approved products alludes to the potential barriers faced by these cutting-edge therapies.

To facilitate the widespread adoption of cell based therapies, the barriers must be identified and overcome. The delivery of cell based therapies to consumers on a global scale comes with the challenges of reproducibly manufacturing, transporting, and administering these treatments to thousands of patients (1). Regardless of whether ongoing clinical trials demonstrate efficacy, the availability of these treatments beyond the clinical trial environments may be hindered by the simple inability to deliver cells in adequate numbers to prospective patients in a timely fashion. Logistical considerations, the ever increasing price of consumables, production time, reimbursement schemes, geopolitical factors, and lack of expertise are all hurdles that we need to overcome to facilitate widespread adoption and implementation of cellular therapies in Europe. In this article we discuss the hurdles to therapy with ATMPs in Europe with a specific focus on regulatory T cells (Tregs). Development of Tregs as a therapeutic medicinal product has seen increased interest in the last decade, but ongoing research has highlighted potential barriers to adoption as a therapeutic product. Some of these issues are specific to Tregs due to the complexity of their manufacture, while others relate to the broader cell and gene therapy (CGT) space.

## Treg cell therapy as a therapeutic agent

The interest in the therapeutic potential of FOXP3-expressing CD4^+^ Tregs has seen multiple use cases examined in animal studies, with adoptive transfer of Tregs being used to treat arthritis (2), allergic airway inflammation (3), and graft rejection in transplantation models (4). The use of Tregs has been under investigation in human based trials for over a decade. The outputs thus far are focused mainly on early stage clinical trials, with the first results being published by Trzonkowski et al. (5), and subsequent early clinical trial data in 2010 by Brunstein et al., Di Ianni et al., and Desreumaux et al. (6–8). In the report on their phase I/IIa trial, Treg infusion was found to be safe and well tolerated (8). Subsequent reports in 2014 demonstrated the safety of *ex vivo* expanded autologous Tregs for the treatment of T1DM (9–11), with some evidence of increased pancreatic islet survival and decreased exogenous insulin requirements in the Treg-treated group (9). There is abundant preclinical data to suggest a role for Treg therapy in preventing rejection following allotransplantation (12). The promise shown in these preclinical studies has led to clinical trials examining their potential role in preventing transplant rejection, a need currently met by lifelong immunosuppressive drugs that come with significant side effects and morbidity. Phase 1 clinical trials have reported on the safety of *ex vivo* expanded autologous Tregs in kidney transplantation. These studies demonstrated the safety of the therapy as well as the proof of concept of expanding autologous Tregs *ex vivo* and re-infusing them into recipients (13–16). There are ongoing Phase 2 clinical trials which look to demonstrate the safety and efficacy of Tregs in maintaining tolerance in living donor kidney transplant recipients (17).

To date over 50 clinical trials of Treg therapy have been completed or are ongoing according to www.clinicaltrials.gov. The initial results have demonstrated the safety of adoptive transfer of Tregs, and certain studies have reported early efficacy data, albeit in very small numbers of patients. However, we are yet to see large scale Phase 3 data to support the use of Tregs in immune-mediated pathologies.

## Barriers specific to Tregs

The manufacture of traditional pharmaceuticals is based on batch processing, in which large volumes of drug can be produced in centralized specialized centers. This “off the shelf” approach results in the availability of large amounts of therapeutics, produced at scale, which facilitates optimization of production strategies to be as economic as possible. Currently, however, the same manufacturing pathway does not exist for ATMPs such as autologously-derived Tregs. Instead of a single product targeting a wide patient demographic with a given clinical condition, ATMPs target very specific groups of patients or individuals, making rapid, efficient commercial production extremely difficult. Traditional chemical-based compounds can be produced in a single center and distributed globally, usually with little concern over sell by dates or product spoilage. Most are stable at room temperature and can be stored relatively simply prior to use, and administered with little difficulty by untrained personnel, including by the patients themselves. ATMPs, however, are live products, and require specialized, and thus expensive, transport and storage networks, and have limited shelf life. Coupled with their method of administration and their patient-specific action, they present a number of novel challenges when compared to more traditional medicines. Clinical trials involving the use of Tregs are also subject to a stringent approvals process, in keeping with ATMP legislation in the EU. Furthermore, any investigational medicinal product (IMP) that is undergoing a first in human trial, is also required to submit an investigational medicinal products dossier (IMPD). According to the EU directive 2001/20, the dossier must provide information on aspects such as manufacturing quality, toxicological and pharmacological data, trial protocol and any prior clinical trial data. The preparation of these dossiers is a costly and time consuming process, with dossiers often falling short of quality requirements (18).

## Source of Tregs

One of the first decisions made when designing a cell based therapy is whether autologous cells, derived from the intended recipient, or allogeneic cells, derived from a 3rd party donor, are to be used. Currently, the majority of studies examining adoptively transferred Tregs use autologous cells, which are isolated from the patient, expanded *ex vivo*, and then re-infused (19). In other studies in haematopoietic stem cell transplantation (HSCT), both autologous cells, occasionally from the same donor as the HSC, and allogeneic Tregs have been utilized to prevent graft versus host disease (GvHD), with the allogenic cells undergoing human leukocyte antigen (HLA) matching to ensure at minimum four alleles match between the donor and recipient (7, 20). Whichever method is chosen, the production of adequate cells for therapeutic effect is a time consuming and costly procedure when compared to traditional pharmaceuticals.

While autologous cells are the chosen therapy in many clinical trials which examine Treg adoptive cell transfer (ACT), this presents many challenges. The manufacturing process must be reproducible and must ensure an adequate number of cells are produced to allow effective treatment. This can be difficult, especially in cases where the cells are intended for use in immunocompromised individuals. There have been several reports of studies in which Tregs isolated for autologous transfer failed to meet minimum numbers necessary (6, 10, 20). The isolation of leukocytes, their subsequent purification, expansion and reinfusion is complex and costly. An ideal solution would be if large numbers of pre-processed cells were available for infusion at short notice into individuals requiring cellular therapy.

To facilitate this “off the shelf” approach, non-autologous third-party derived Tregs would be required. This could potentially involve the use of allogeneic Tregs which have undergone an HLA matching process, potentially providing a short term immunosuppressive effect without the need to expand and re-infuse autologous Tregs (21, 22). However, unmodified non-autologous Tregs would likely be quickly rejected by the recipient’s immune system, resulting in a very expensive and short lived therapeutic effect. To ensure efficacy of non-autologous Treg therapy, the cells would need to undergo modification to prevent their rejection. Genetically engineered Tregs which have undergone deletion of β-2 microglobulin and class II major histocompatibility complex transactivator (CIITA), thus eliminating major histocompatibility complex (MHC) I and MHC II antigen expression, may avoid allorecognition and facilitate survival and efficacy of the adoptively transferred cells. The absence of MHC I, however, may result in the destruction of the modified cells by recipient NK cells due to the “missing self” hypothesis (23, 24). Furthermore one could speculate that the absence of MHC expression may potentially alter Treg function *in vivo*, with studies suggestion MHC expression is important for the immunosuppressive effect of Tregs (25). Such strategies have been attempted with success in allogenic cell transfer models, including HSC and Treg adoptive transfer (26, 27). In these studies depletion of β2-microglobulin resulted in cells which avoided allorecognition and thus Treg and NK cell cytotoxicity. Additional modifications to the allogenic Tregs could potentially inhibit NK mediated killing of MHC I deficient cells, with research suggesting that overexpression of HLA-E or CD47 could prevent NK cell mediated cell death (28–30).

## Isolation of cells

Unfortunately, there are no exact markers for either polyclonal or antigen-specific Tregs, as such isolation of cells is a balance between selection of a sufficiently pure population of cells, while avoiding unnecessarily exclusion of large volumes of useful material. The method of isolation of Tregs for use in human trials provides an ongoing obstacle which must be overcome to allow the widespread production and manufacture of Treg based therapeutics. The process is labor intensive and costly, requiring specialized equipment and significant experience in good manufacturing practice (GMP) manufacturing and quality control (31).

There are numerous methods by which Tregs can be isolated, with significant regulatory and cost differences between methods. Many groups opt to use magnetic sorting devices which isolates cells using ferromagnetic-conjugated antibodies to cell surface markers of interest. The benefits of these magnetic sorting systems is that they are closed systems, and the reagents and protocols are all CE validated which facilitates their GMP certification. Early studies which isolated Tregs using these methods and utilized the cells as GvHD prophylaxis showed promising results (7, 32). While these systems provide a GMP compliant method of bulk cell sorting, they struggle in differentiating between high and low expression of CD molecules. In the case of Tregs for example, magnetic systems often result in contamination of CD25^high^ Tregs with CD25^low^ TConv (33). Thus, the magnetic sorting systems provide a sterile GMP compliant method of debulking the initial input and removing many unwanted cell types, but still struggle to produce a high purity Treg output.

Flow cytometry sorting offers benefits over magnetic sorting as it permits more precise identification and isolation of cells based on varying expression of CD25 as well as numerous other surface markers. In fact, isolation of CD25 high populations produces Tregs with greater functional properties where immunosuppression and immune regulation are concerned (34, 35). Fluorescence-activated cell sorting (FACS) offers robust high purity cell isolation, however, this method presents a significant regulatory challenge in Europe, as outside of very specific conditions, flow cytometry based cell sorting under GMP conditions is not readily available (36). EU countries require adherence to GMP guidelines for all cellular products (Directive 2003/94/EC and its Annex 2), notably placing importance on batch-to-batch consistency. Because of the nature of droplet based sorters, maintaining sterility is of major concern, and traditional flow cytometry was not designed with sterile purification in mind (37–40). Furthermore, the cytometer itself has numerous parameters which are modifiable, such as flow rate and detector gain, and daily variations in these parameters even in the absence of recalibration can result in inconsistencies. GMP compliance regulation is stricter in Europe than in the US, as even phase 1 clinical trials require full GMP adherence, which is not the case in the US (36). It is clear that there are many benefits to FACS as an isolation technique, including purity and ability to select specific Treg subpopulations, but until GMP compliant systems become more readily available, FACS sorted Tregs approved for human use will be out of reach for all but the most specialized of centers. To date only five groups have received regulatory approval for the use of FACS sorted Tregs, the Trzonkowski group in Poland, the Bluestone group in San Francisco, the Edinger group in Germany, and more recently in the UK, the Lord group has received approval for the use of FACS-sorted Tregs for the treatment of Crohn’s disease using the Miltenyi MACSQuant Tyto (41), and in France the Dumont group utilized a SONY FACS based system for CAR-Treg use in solid organ transplantation (42).

Other methods of cell sorting have been investigated in the production of Tregs for clinical use. Microfluidics chip-based sorters such as the Gigasort system, which provides a relatively high throughput closed sorting system, have shown promise as high throughput isolation methods (43). Studies have shown that modifications to the isolation procedure, when performed in a level 3 clean room environment, can isolate Tregs reliably in conditions which would satisfy GMP requirements (43). A similar process of isolation is closed cartridge super vast valve sorting, which uses a microchip based magnetic valve to select cells of interest in a closed sterile environment (33). As with the microfluidics system, it is chip based and single use, which allows conformity to necessary GMP standards for production of ATMPs.

A further method of isolation has been explored which somewhat circumvents regulatory issues surrounding ATMPs. Streptamer-based technologies involve loading Fab portions of antibodies to antigens of interest onto streptacin cores. These cores are then conjugated to magnetic beads, allowing highly specific isolation of cells of interest. The key difference to other isolation methods is following isolation of the cells, the addition of D-biotin allows a complete dissociation of the streptacin-antibody complex from the cell of interest (44). This produces cells which are by definition “unmodified” in the isolation process, meaning they are not classified as ATMPs for regulatory purposes. Thus, in instances where small numbers of cells are required without expansion, streptamer based technologies may provide a solution which avoids excessive regulatory oversight. However, would one seek to further modify or expand these cells *ex vivo* they once again become classed as ATMPs, and as such are subjected to all the relevant regulatory frameworks.

Once cells have been isolated and expanded, the method of storage while they await transportation and infusion into the patient is a topic for debate. While many centers opt for refrigeration or ambient temperature storage, there are groups which prefer cryopreservation of Tregs to facilitate transport and storage of cells.

## Storage of cells

An important issue regarding Treg use in clinical trials is to decide whether to use fresh or cryopreserved cells. As the cell product must be prepared in a GMP clean room laboratory which may be geographically distant to the treatment site, it would be easier to use cryopreserved cells for logistical reasons. In addition, storing cells prior to the infusion allows the clinical team to choose the best timing of therapy, which is particularly important in solid organ transplantation, as well as apply therapeutic schemes including multiple administration regimens. However, cryopreservation can have an impact on Tregs. Therefore an optimal protocol for cell cryopreservation is required (45).

The simplest method is to freeze donor peripheral blood mononuclear cells (PBMCs) and to then isolate and expand Tregs after thawing, thus also delaying the complex GMP manufacturing cost to a time when infusion is confirmed. However, available data shows limitations of such an approach. For example, Elkord performed a flow cytometric analysis of fresh and frozen-thawed PBMCs from the same donors and found that cryopreservation significantly reduced Treg frequency (46). Another study, which included healthy subjects and an additional group of HIV patients, confirmed a reduction in Treg numbers after cell freezing in both groups (47). The authors propose an alternative explanation to their observations, concluding that cryopreservation may influence the detection of the surface proteins and therefore modulate the detection of Treg markers. On the other hand, other groups did not observe Treg reduction after PBMC freezing (48, 49). However, it is worth noting that freezing protocols and media vary between groups.

Another approach is to freeze freshly isolated Tregs or expanded cells. Peters et al. (45) showed that CliniMACS isolated Tregs could survive cryopreservation in liquid nitrogen, but their suppressive activity was decreased after thawing. However, it could be restored after Treg stimulation and expansion. They also report that they did not find a decrease in the suppressive activity of Tregs expanded before freezing. Modifications to the freezing and thawing protocol have also been examined to determine if they impact Treg recovery, viability and phenotype. Kaiser et al. (50) experimented with a freezing medium with reduced DMSO concentration and found that 5% DMSO facilitated enhanced Treg recovery and viability. Gołab et al. (51) analyzed two strategies of cell biobanking for Treg therapies: CD4^+^ cells cryopreserved for further Treg isolation and expansion, and Tregs (CD4^+^CD25^hi^CD127l^*o*/–^ cells) frozen after expansion. They observed a high percentage of apoptotic cells after thawing and poor cell recovery after overnight culture. Tregs were more sensitive to cryopreservation as the percentage of early apoptotic cells was double that of thawed CD4 + cells. Notably, the percentage of necrotic cells was similar in both groups. They also found a decrease in the percentage of CD4 + CD25hiCD127− and CD4 + FoxP3^+^ cells obtained from both cryopreserved groups of cells. However, the authors could distinguish Tregs that were thawed then expanded, from Tregs that were expanded, frozen and then thawed. Both sets of Tregs passed a quality and purity test based on FDA requirements for cell products. The analyses included microbiological purity and endotoxin and mycoplasma detection. The cells contained less than 5% B cells, their viability was more than 75%, there were more than 90% CD4 + and 60% FoxP3 + cells, and the remaining contaminant expansion beads were less than 100 beads per 3 million cells. Finally, Tregs from both groups showed good suppressive properties. These results suggest that using cryopreserved Tregs is possible, but they may require additional expansion after thawing. Moreover, the analysis of Tregs cryopreserved in one study (NCT02129881) showed that after 12 weeks, thawed cells from healthy donors and recipients showed >75% viability and cell recovery was >90%. What is more, the average purity (staining of FOXP3, CD4, and CD25) was >70% in both groups, and the cells maintained their suppressive functionThese results are encouraging for clinical application of cryopreserved T regulatory cells (52).

## Antigen specific Tregs

While there is emerging evidence from pre-clinical and clinical data that adoptive transfer of Tregs is a promising therapy for autoimmune diseases and transplant rejection, these studies have been performed with polyclonal Tregs. The notable exception to this is the ONE study, in which two centers utilized donor reactive Tregs to modify the immunosuppressive regime in kidney transplant recipients (15).

There is increasing evidence that antigen specific Tregs provide superior immunosuppressive function, with minimal off target effects (53–57). These studies demonstrated that the antigen specific Tregs can offer potent immunosuppressive activity in a disease specific manner. This target effect also occurs with far fewer cell numbers than necessary in polyclonal Treg based experiments. The increased efficacy of these antigen specific Tregs may be due to superior tracking of Tregs to the site of interest due to antigen directed migration (58, 59). However, antigen specific Tregs are not a simple solution, as isolation, purification and expansion of antigen specific Tregs is a complex process, largely due to the precursor frequency of cells of the required specificity in peripheral blood (60). Genetic engineering of antigen specific Tregs might provide an alternative solution, as it would confer antigen specificity to a larger population of isolated Tregs rather than focusing on expansion of existing antigen specific Tregs. However, this method still has drawbacks, as the process of genome editing Tregs might also result in genetically edited antigen specific Tconv, which may have a proliferative advantage over Tregs, especially in cytokine rich media commonly used for Treg expansion (23).

## Stability and plasticity

One of the remarkable properties of Tregs is their plasticity, with research showing that even specific subsets of Tregs can have very heterogenous phenotypes. Data have shown for example that Th17 cells can range from pathogenic to regulatory phenotypes, regardless of their initial stimulus (61, 62). Similar plasticity is seen in Tregs, with an ability to phenotypically mimic both inflammatory and regulatory Treg subsets (63). Mechanistically, this plasticity is due to the ability of Tregs to express different master transcription factors, which allow them to adopt distinct lineages and localize to specific tissues under specific conditions. This plasticity is a key feature of the *in vivo* action of Tregs as a component of the adaptive immune system, but in considering the role of adoptively transferred cells, carries risks.

Inducible plasticity could potentially serve as a mechanism of targeting specific tissues or inflammatory conditions. Forced expression of specific chemokine receptors such as CXCR3 or CCR5 could increase the efficacy of Tregs used to treat MS or T1DM (64, 65). However, this plasticity has also been a cause of concern in the development and regulation of Treg based cellular therapies. Tregs can take on a pathogenic phenotype in certain disease states, suggesting feedback from certain disease related factors can induce phenotype switching from an anti-inflammatory phenotype to a pathogenic Th like phenotype (66). Multiple methods have been proposed to increase the stability of Tregs *in vivo*, the details of which are beyond the scope of this article [see (19) for a comprehensive overview].

To validate the safety of Tregs, regulatory authorities have to be confident that once the product is infused into a patient, it retains the properties which the approved product is licensed for. As such, demonstrating stability will continue to be a key part of the development process for Tregs. Utilizing methods such as FOXP3 overexpression, IL-2 induced persistence, or synthetic receptors only adds to the complexity and cost of designing and implementing Treg based therapies.

## Regulatory environment

**FIGURE 1:**
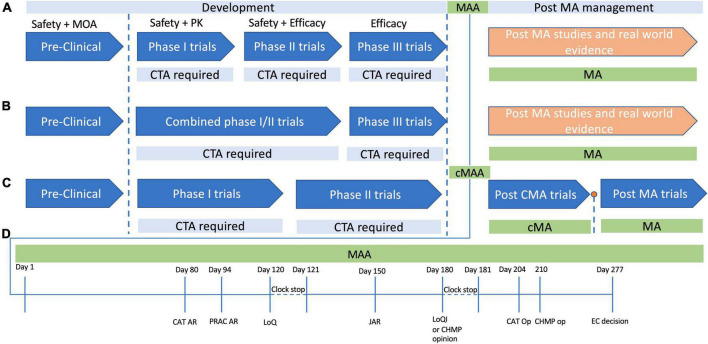
Development and approval pathways for advanced therapy medicinal products (ATMPs) in Europe. The development cycle of products for human use has a number of steps which ensure the safety, efficacy and quality of the product in question. **(A)** In typical product development cycle, pre-clinical research is followed by first-in-human or Phase 1 clinical trials, with Phase 2 assessing safety and early efficacy, and Phase 3 providing confirmation of efficacy. In each case a clinical trial application (CTA) is required before each phase. **(B)** It is often not possible to perform healthy volunteer trials with ATMPs for various ethical reasons, and as such there may be combined phase 1/2 trials in which safety and efficacy are tested simultaneously. Phase three studies then provide data to support a marketing authorization application (MAA), and if marketing authorization (MA) is granted, then post MA trials are usually performed to confirm efficacy and safety further, with continued MA often dependent on follow up data. **(C)** A conditional MA (cMA) approval process exists where there is an unmet clinical need and a belief that the ATMP may provide benefit to the patient cohort even in the absence of comprehensive data. A cMA may be granted, in advance of Phase 3 trials, and following convincing data a full standard MA may be approved, or MA may be withdrawn if there is not convincing evidence to support the products approval. **(D)** The MAA review process typically takes place over a timeline of 277 days committee for advanced therapies. At day 80 and day 94 the committee for advanced (CAT) and the pharmacovigilance risk assessment committee (PRAC) generate their assessment reports, which are then consolidated with the committee for medicinal products for human use (CHMP) assessment points into list of questions (LoQ). The assessment clock then stops and the submitter has 90 days to respond to the LoQ. After the 90 days, or whenever the applicant submits their responses, the CAT, CHMP, and PRAC generate a joint assessment report (JAR), which is then sent to the applicant to view, and is formalized at day 180. At this stage if all issues from the LoQ are addressed, they may produce a draft opinion, or otherwise they will present the applicant with a list of outstanding issues (Looi). The then have another 90 days clock stop to address any remaining issues, following which the CAT and CHMP produce their opinions, and ultimately the European centralized committee produces their decision on day 277.

The development process for Treg based therapies is subject to the regulatory approvals process required for ATMPs, with few exceptions (Figure 1). As such the journey from preclinical studies to approval for clinical use can be a long and costly process. A 2021 study by Iglesias-Lopez et al. (67) was one of the first to examine and compare the approval process in the European Union (EU) and in the United States of America (USA). While the study concluded that there were similar barriers in place for the approval of gene and cell based therapies in the USA and EU, the attainment of these milestones had some important caveats which may make product development more difficult in Europe compared to the US except in certain circumstances. At the time of publication, the study identified that 15 ATMPs had been approved in the EU, with 9 being approved in the US (67). Of the 15 approved in the EU, 10 had orphan drug status, compared to 5 of the 9 approved in the US. The use of orphan drug status facilitates rapid approval of these therapies, as there are procedures and frameworks to expedite the development for therapies for orphan diseases. However, a significant regulatory difference exists between the US and the EU where orphan therapies are concerned. In the US therapies for orphan diseases do not need to demonstrate significant benefit over standard of care, allowing approval for therapies which target orphan diseases without a significant hurdle of proving superior efficacy. Currently this is not the case in the EU, as even therapies for orphan diseases must demonstrate significant benefit over standard care (68, 69). The study by Iglesias-Lopez et al. (67) identified that over half of the approved therapies in Europe had the “unmet clinical needs” designation, meaning they were targeting conditions for which there was no current therapy. While this designation facilitated market approval for these ATMPs in Europe, it also highlights a significant hurdle for cellular therapies in Europe. If all current and future therapies for non-orphan diseases need to overcome the significant benefit threshold to allow approval, one can imagine that it will hinder the development of cell based products in Europe. If early trials are not suggesting there is a clear benefit to a cellular therapy over conventional therapy, pharmaceutical companies or other funding agencies may be reluctant to pursue further trial stages if there is a risk the significant benefit threshold will not be met, and approval will not be granted.

The are other regulatory hurdles which are specific to the European market. Where the FDA publishes guidelines and regulations for the entirety of the United States, the EMA and medicinal product regulations in Europe cover different jurisdictions and regulatory authorities. The marketing authorization application process is a centralized procedure managed by the EMA, and has the benefit of having a singular evaluation process (70, 71). However, the approval of clinical trials utilizing ATMPs, as well as monitoring and evaluation of these trials, is devolved to the individual national competent authorities (NCAs). In the UK for example to undertake a clinical trial with a cell therapy such as autologous Tregs, the donation, procurement and testing of the cells must meet the conditions of the European tissues and cells directive (2004/23/EC). In the UK the competent authority for ensuring adherence to these standards is the Human Tissues Authority (HTA). Once the cellular components have been made available under the tissues and cells directive, legislation for medicinal products now applies as per HTA and MHRA regulations. While stringent controls are necessary to ensure safe development of these novel therapies, the application of these criteria across multiple individual nations within the European region adds significant complexity. In fact, entities who are involved in developing ATMPs in Europe cited meeting country specific regulatory requirements as one of the major barriers to undertaking trials in Europe (72).

This difficulty is particularly present in cases where products have undergone any form of genetic modification, as they then must comply with the genetically modified organism (GMO) legislation. The GMO legislation in Europe was originally designed for the food and agriculture sector but was expanded to include medicinal products such as cell based therapies (73). These regulations were drafted by the European commission but intended to be implemented and enforced on a national level. ATMP developers have remarked that differing interpretation and implementation of these guidelines by separate member states has made compliance with guidelines confusing and very costly (72). Such is the variation in national authorities implementation of guidelines, that developers have stated they often select regions for trials based on local knowledge of ATMPs and perceived ease of development compared to other regions. This itself should be cause for concern as there will be a huge population bias in clinical trial data from within Europe if all studies are performed in locations with relatively easier approvals processes. These delays and hurdles to stakeholders make the EU a less competitive environment for large scale multicentre trials compared to the USA, where approval can be sought on a national scale through the FDA and applied to the huge and varied population. Such is the environment in Europe, a large consortium of stakeholders has called for a centralized approach to ATMPs exempting them from the national regulations and instead moving to a central European approvals process (74). This vision has been somewhat implemented in the pursuit of treatments for SARS-CoV2, with temporary exemptions provided to aid the development of novel therapeutics to assist in the pandemic. This emergency deregulation facilitated rapid multicenter investigations of potential therapies for COVID-19, and this is a framework developers hope to see expanded to ATMPs generally (75). There has been increased support for a shift to a life cycle HTA assessment, with conditional approval occurring more rapidly, with regulators potentially using post approval real world evidence (RWE) and a dynamic approval model to facilitate quicker approval, with feedback from patient data informing ongoing approvals decisions (76, 77).

Another source of concern is the regulatory environment in a post-Brexit EU. The UK constitutes a large proportion of ATMP development in Europe, with nearly 25% of European ATMP developers based in the UK, and over 70 active companies investigating ATMPs (78). There is uncertainty around how exactly UK regulation may change in the years after Brexit, and whether this will add increased complexity to the already nebulous EU regulatory environment. Currently the UK government has confirmed that it will remain closely aligned with the EU clinical trials regulation (CTR) (79). While this is somewhat reassuring, this pertains to laws already implemented by the EU, and does not have provision for legislation which has not yet been implemented. Given the rapidly evolving nature of ATMP regulations, if the UK chooses to diverge from the EU CTR as it is updated, or fail to update its policies in a timely fashion, research groups in the UK may see their participation in multi-center EU trials severely limited.

## The cost of GMP compliance

In Europe, the development of ATMPs such as Tregs must adhere to GMP guidelines at all stages of the development process. As such GMP grade materials are required throughout the entire production process (80). However, a study which surveyed producers of ATMPs in Europe determined that manufacturers often have difficulty in sourcing materials which are GMP grade, and when they are available, they can prove extremely expensive (72). There have been several cases in the literature which have noted difficulties in ATMP manufacturing which included manufacturing complexities, quality control of reagents, and cost (81–83). These effects are often compounded by the approvals process required to make the reagents GMP compliant. Acquiring approval for a product to be used in GMP process is a time consuming and costly process, which often places it solely in the realm of the larger manufacturers. In Treg production for example, there are many products for which only one supplier in Europe has been approved for GMP grade production. When this producer is based in a European country this can make acquiring process-specific reagents in a timely fashion extremely difficult in a post-Brexit UK. While some larger enterprises involved in the ATMP development space have partly solved these issues by producing their own in house GMP grade reagents, this is not financially or technically feasible for many of the smaller operations. This is concerning given over 65% of the companies involved in the European ATMPs market are small to medium sized enterprises (SMEs), suggesting that a large proportion of the sector may have difficulties in accessing sufficient resources for production. Similar issues were highlighted in a 2013 study which highlighted that SMEs often face more regulatory difficulties and supply chain issues compared to large multinationals (84). As with the previously discussed regulatory differences between NCAs, GMP legislation and standards are interpreted and applied differently across different member states, further compounding the complexities in ensuring adherence to guidelines. This fact was highlighted by research produced by AGORA, an EU funded consortium investigating solutions to problems in the GMP sector. They determined that confusion due to significant heterogeneity in the application of regulations across EU member states is “creating a severe barrier to the development and delivery of ATMP medicines” (85).

The GMP grade reagents and materials are one part of the manufacturing process, but expertise in GMP manufacturing is another, unfortunately scarce, resource. In a report by Mckinsey Company (87) examining the outlook for cell and gene therapies in Europe, the consulting group reflected on the dearth of experience and manufacturing expertise in Europe when compared to the USA. The Atlantic divide in CGT success is obvious when looking at the output, with only 13 percent of launched CGTs coming from Europe (86). This divide is likely to widen in the future, with industry experts estimating that the two largest plants for CGTs in the US will soon outstrip the entire capacity for manufacturing in Europe (87). The US centric environment for manufacturing of CGTs further compounds the problem in Europe, as European scientists are drawn toward North America with the promise of higher wages and better opportunities for advancement in the ever-expanding US life sciences industry. This often results in remaining EU experts gravitating toward smaller hubs of leading life science companies and universities in the UK, Switzerland, France and Sweden.

Acquiring the necessary personnel, GMP grade reagents, equipment which meets the criteria for GMP grade production, and facilities in which to produce the cell product is part of the very costly barrier to entry to Treg manufacturing. Once all of these aspects are in place however, significant hurdles still exist, not least the actual production of the Tregs themselves. Treg production in its current format is extremely labor intensive and complex, and as a result the turnaround times are significant.

## Logistical issues

The European shortage of manufacturing expertise and capacity is further hampered by the logistical issues of manufacturing in Europe. Many cell and gene therapy products are transported unfrozen, under strict temperature controls. As such they have a short expiry window and the manufacturing process, transportation, and elective administration in the clinical environment must all be streamlined and precise to ensure the cells reach their intended patient at the right time (88). However, this “just-in-time” delivery process has encountered severe disruptions in Europe in the wake of both COVID-19 and Brexit.

A McKinsey (89) report on the impact of COVID-19 on CGT manufacturing in Europe highlighted the difficulties and delays cell therapy manufacturers were having as a result of disruption due to the pandemic. A roundtable with the executives of 20 CGT companies uncovered that 22% were facing difficulties in supply procurement, 11% were having difficulties with product shipping, and 33% stated issues with significant delays to manufacturing, or manufacturing being completely halted. Furthermore, the research and development of new CGTs was also significantly impacted, with 55% or the respondents stating that site activation for trials was paused and patient recruitment was halted, and 55% stating that there were severe disruptions in follow up appointments of enrolled patients. The report suggests that the pandemic related disruptions will continue, with 63% of companies reporting development delays greater than 6 months, with 18% of those claiming they had delays of up to a year. These delays and logistical challenges result in excess spending and budget issues, with an estimated 43% of companies surveyed needed additional unexpected capital injections to facilitate continued development and production of their CGTs (89).

While there is little published evidence currently on the direct impact of Brexit on life sciences manufacturing, it is likely that the supply chain issues, product shortages and workforce issues seen elsewhere in the UK post-Brexit have also affected the life sciences industry. A report produced by Coleman Parks, a strategic business research firm, suggested up to 66% of UK based companies are enduring delays and longer lead times for starting materials, rising to 79% where the healthcare sector is concerned (90). While there has been no formal data published so far, it is difficult to imagine that Britain leaving the EU has had a positive effect on cross border transportation of goods.

## The cost of ATMPs

There are 23 ATMPs with current marketing approval within the EU (Table 1), with the most recent approval being Roctavian, a gene therapy product used to treat severe hemophilia A (91). There is an expectation that in the next decade a large number of ATMPs will gain MA, and that globally the industry will be worth £9-14 billion annually by the middle of the decade. With this advancement in medical therapy comes the question of how much such therapeutics will cost, and who pays. Currently ATMPs are extremely expensive, and as such in the UK for example they have only been granted authorization for use after other lines of treatment have failed, or if there is no viable alternative (92). With costs such as £282,000 for a single infusion or Tisagenlecleucel, £594,000 per course of treatment with Strimvelis, and nearly £1 million pounds for Zyntelgo (autologous CD34 cells for beta thalassemia), Treg therapies are likely to cost in the order of tens to hundreds of thousands per dose. As such, there are difficult decisions to be made regarding perceived value for money where ATMPs are concerned, the budget implications of these treatments, and potential methods of cost recovery.

**TABLE 1:** Approved advanced therapy medicinal products (ATMPs) in Europe.

Name	ATMP type	Function	Authorization date	Orphan status?	Notes	
Chondrocelect	TEP	Autologous chondrocyte implantation for the treatment of cartilaginous lesions in the knee	5/10/09	No	MA withdrawn in 2016	NCT00414700
Glybera	GTMP	Adenovirus vector delivery of human lipoprotein lipase to treat LPL deficiency	25/10/12	Yes	MA not renewed (MA ended 2017)	NCT03293810
MACI	TEP	Matrix induced autologous chondrocyte implantation for repair of cartilaginous defects in knee	27/06/2013	No	MA ended June 2018 (not renewed)	NCT00719576
Provenge	CTMP	Stimulation of T-cell immune response against prostatic acid phosphatase in prostate Ca	06/09/2013	No	MA withdrawn in May 2015	NCT00065442
Holoclar	TEP	Autologous corneal epithelial cells for use in Limbal stem cell deficiency		Yes		NCT02577861
Imlygic	GTMP	Genetically engineered herpesvirus use to treat melanoma	16/12/15	No		NCT00769704
Strimvelis	GTMP	Autologous CD34 + cells transduced with ADA cDNA used to treat ADA-SCID	26/05/16	Yes		NCT03478670
Zalmoxis	CTMP	Adjunct therapy in HSCT therapies for hematological disorders	18/08/16	Yes	MA withdrawn Oct. 2019	NCT00914628
Spherox	TEP	Autologous chondrocyte transfer for cartilage defects	10/07/17	No		NCT01222559
Alofisel	CTMP	Allogenic adipose derived stem cells for treatment of complex peri-anal fistulae	23/03/18	Yes		NCT03706456
Yescerta	GTMP	CAR-T cell therapy for NH lymphoma	23/08/18	Yes		NCT02348216
Kymriah	GTMP	CAR-T cell therapy for Acute lymphoblastic leukemia	23/08/2018	yes		NCT02435849
Luxturna	GTMP	AAV transfer therapy for inherited retinal dystrophy	22/11/18	Yes		NCT00999609
Zynteglo	GTMP	Autologous CD34 + cells transfected with human βA-T87Q-globin gene to treat Beta Thalassemia major	29/05/19	Yes	MA withdrawn March 2022	NCT01745120
Zolgensma	GTMP	Adenoviral gene transfer therapy for SMA-1	18/05/20	Yes		NCT02122952
Libmeldy	GTMP	Lentiviral based gene therapy for metachromatic leukodystrophy	17/12/20	Yes		NCT01560182
Tecartus	GTMP	CAR-T cell therapy for mantle cell lymphoma	14/12/20	Yes		NCT02601313
Skysona	GTMP	Lentiviral transduced HSC for treatment of cerebral adrenoleukodystrophy	16/07/21	Yes	MA withdrawn Nov. 2021	NCT01896102
Abecma	GTMP	CAR-T cell therapy for multiple myeloma	18/08/21	Yes		NCT03361748
Breyanzi	GTMP	CAR-T cell therapy to treat B cell lymphoma	04/04/22	No		NCT03575351
Carvykti	GTMP	CAR-T cell therapy for treatment of multiple myeloma	25/05/22	Yes		NCT03548207
Upstaza	GTMP	AAV2 based Gene therapy of aromatic L-amino acid decarboxylase (AADC) deficiency	PO May 2022	Yes		NCT01395641
Roctavian	GTMP	AAV5-Factor VIII gene therapy for Hemophilia A	PO June 2022	Yes		NCT03370913

Outline of all ATMPs which have been approved for use by the EMA since the inception of legislation for ATMPs. Some listed have since been discontinued due to unfavorable outcomes in later stage clinical trials which resulted in market authorization (MA) being withdrawn by the EMA. TEP, Tissue engineered produce; GTMP, Gene therapy medicinal product; CTMP, cell therapy medicinal product; AAV, Adenovirus associated vector; CAR, Chimeric antigen receptor; PO, Positive opinion (pertaining to EMA approvals process, usually indicates market authorization is to follow).

The willingness of healthcare systems to pay the cost of treatments is traditionally determined through HTAs, usually through the calculation of a set cost/effectiveness ratio the provider is willing to accept. In the UK for example, NICE has traditionally used an Incremental Cost Effectiveness Ratio (ICER) of between £20-30,000 per quality adjusted life year (QALY) as the range it considers acceptable (93). The first wave of NICE approved ATMPs were appraised under the cancer drugs fund (CDF), which uses different value judgment criteria and as such the NICE ICER threshold is often not adhered to. It is argued that given the new nature of these treatments it is difficult to ascertain the ICER accurately, with higher thresholds being applied as a result (94). There is speculation that ATMPs may be value assessed under a new NICE highly specialized technologies route which may allow for up to £300,000 per QALY in certain cases (95).

## Payment and reimbursement models

To facilitate patient access to ATMPs such as Treg therapies, new methods of pricing and reimbursement may have to be implemented. Traditional usage based pricing models are unsuitable for ATMPs, especially as many involve therapies which are very expensive, and often HTAs only have short term phase 1/2a data assessing their clinical efficacy (96). Further issues arise from the nature of ATMP administration when compared to traditional therapeutics. While traditional drug therapies can be simply discontinued if the patient fails to respond to treatment, thus negating any future cost of therapy, the same is not true of single use ATMPs where the full cost is incurred from the outset. HTAs and the health care systems they represent have outlined their unease at potentially outlaying up to £1 million for a treatment which may not provide benefit to the patient, a risk many feel they cannot justify.

Thus, to make ATMPs and their costs more palatable to health authorities, new pricing models which minimize the risk to the payers have been proposed. Instead of a pay per use model, pricing which linked the outcome to the cost were proposed, which would mitigate some of the risk for payers (97). This concept of outcome based reimbursement (OBR) has gained in popularity over the last 10 years, and it played a key role in the launch of CAR-T cell based therapies Kymriah^®^ (Novartis, Basel, Switzerland) (tisagenlecleucel) and Yescarta^®^ (Kite Pharma, Los Angeles, CA, United States) (axicabtagene ciloleucel) in Europe (98). While an OBR based system places more risk on the manufacturer, it was deemed necessary to incentivize the uptake of novel, extremely expensive therapies. The advantages are relatively straightforward however, as the payer is protected from the cost of a failed treatment, and the manufacturer gaining increased market exposure due to the perceived assurance of efficacy. Currently in the UK and France these therapies are being reimbursed using a Coverage with Evidence development scheme (CED), in which longer term outcomes of treatment will be used to inform future pricing. In the UK this CED has been provided through the CDF scheme, to allow quicker access to these medications for cancer patients, but it remains to be seen how this pricing and reimbursement model will function in the case of Treg therapies which are not targeting cancer. The payment method is different in Germany, Spain and Italy, with reimbursement being based on individual patient data, as opposed to cohort based data as in the UK and France. There are benefits and downsides to both, as cohort schemes may be cheaper for payers overall when compared to individual data reimbursement schemes. However, should the therapy prove ineffective, individual outcome based schemes will benefit as the payers are unlikely to overpay, whereas in cohort schemes such as the UK, the reimbursement will have already been agreed upon pending the collection of future data (99).

Manufacturers have been eager to development payment options that are agreeable to healthcare systems, to encourage uptake of the treatments and to act as a proof of concept of alternative funding models for ATMPs. In Germany Novartis arranged an outcome based payment system for Kymriah based on patient survival. This agreement was accepted by many of the larger insurance groups in Germany resulting in 60% of the 70 million citizens covered by German statutory health insurance having access to the therapy (100). In the USA Novartis also have an outcome based agreement, where reimbursement is based on patient response at 30 days post initiation of treatment (101). However, the proposals were not universally successful, with the Centre for Medicare and Medicade service (CMS) rejecting the outcome based payment plan. The CMS evaluated the two CAR-T cell therapies and determined that there was insufficient long term outcome data, as well as insufficient current patient numbers to support the proposals (102). They instead opted to adopt the more traditional method of up-front discount pricing, which provides a more attractive price for the payers, but results in a less desirable situation for the manufacturers. Providing discounts rather than outcome based pricing results in increased financial risk for the developers.

The need for new and innovative payment methods to accompany the development of ATMPs is evident. If cell-based therapies such as autologous or allogenic Tregs are to reach mainstream use, the mechanisms of procurement and reimbursement needs to be palatable to the payers. Currently the expense associated with these cellular therapies is a major hurdle, and while approval has been granted in many different health care systems, often it is for unmet needs or disease refractory to other treatment. To facilitate the replacement of current best practice therapy such as pharmacological immunosuppression for transplant tolerance, the proposed cell product will have need to be financially viable as an alternative.

## Discussion

Early phase clinical trial shave shown the promise of Treg therapies, with safety and feasibility being demonstrated, and some encouraging early efficacy data. However, to facilitate large scale adoption and widespread use, there are still significant barriers to overcome.

The production of Tregs for use in clinical applications requires strict adherence to GMP principles with complex isolation, expansion, and preservation processes. As such current production of clinical grade Treg products is concentrated in a small number of specialized commercial producers and large, well-funded academic groups. The importance of the academic centers in the development of ATMPs cannot be overlooked, but the lack of open communication and close guarding of results until they reach the patent or publication stage can result in different lab groups making the same costly mistakes. Certain institutions have recognized this issue, and have proposed moving to an open science model, where all protocols, data, and materials are available for all to see, and a no patenting approach is taken (103, 104). The aim being that open science will encourage collaboration and industry partnerships to foster rapid development of novel technologies and prevent recurring failures due to the lack of availability of negative or unsuccessful data.

While production and storage pose significant challenges, so too does the regulatory environment. The guidelines produced by the EMA and their subsequent interpretation by individual national competent authorities results in regulations that are interpreted and applied differently across the EU. The differences in application of the regulations across the trading block has resulted in hotspots for ATMP development which are seen as more accessible where legislation and compliance is concerned, partially due to local experience and expertise in dealing with the products of interest. This unfortunately has resulted in ATMP development being concentrated in several centers of expertise such as Germany, the UK, France, Switzerland, and Sweden. In order to facilitate larger multi-center clinical trials, the implementation of regulations must become more uniform across the EU member states and their trading partners.

## Author contributions

CH and MD drafted the manuscript. JH and FI reviewed the text, provided corrections and edits, and were involved in the planning of the topics contained within the article. All authors contributed to the article and approved the submitted version.

## References

[1] National Cell Manufacturing Consortium. *Achieving Large-Scale, Cost-Effective, Reproducible Manufacturing of High-Quality Cells: a Technology Road Map to 2025.* (2016). Available online at: https://cellmanufacturingusa.org/sites/default/files/NCMC_Roadmap_021816_high_res-2.pdf (accessed November 1, 2019).

[2] Wright G Notley C Xue S Bendle G Holler A Schumacher T Adoptive therapy with redirected primary regulatory T cells results in antigen-specific suppression of arthritis. *Proc Natl Acad Sci.* (2009) 106:19078–83. 10.1073/pnas.0907396106 19884493 PMC2776462

[3] Xu W Lan Q Chen M Chen H Zhu N Zhou X Adoptive transfer of induced-treg cells effectively attenuates murine airway allergic inflammation. *PLoS One.* (2012) 7:e40314. 10.1371/journal.pone.0040314 22792275 PMC3392250

[4] Issa F Hester J Goto R Nadig S Goodacre T Wood K. Ex vivo-expanded human regulatory T cells prevent the rejection of skin allografts in a humanised mouse model. *Transplantation.* (2010) 90:1321–7. 10.1097/TP.0b013e3181ff8772 21048528 PMC3672995

[5] Trzonkowski P Bieniaszewska M Juścińska J Dobyszuk A Krzystyniak A Marek N First-in-man clinical results of the treatment of patients with graft versus host disease with human ex vivo expanded CD4+CD25+CD127- T regulatory cells. *Clin Immunol Orlando Fla.* (2009) 133:22–6. 10.1016/j.clim.2009.06.001 19559653

[6] Brunstein C Miller J Cao Q McKenna D Hippen K Curtsinger J Infusion of ex vivo expanded T regulatory cells in adults transplanted with umbilical cord blood: safety profile and detection kinetics. *Blood.* (2011) 117:1061–70. 10.1182/blood-2010-07-293795 20952687 PMC3035067

[7] Di Ianni M Falzetti F Carotti A Terenzi A Castellino F Bonifacio E Tregs prevent GVHD and promote immune reconstitution in HLA-haploidentical transplantation. *Blood.* (2011) 117:3921–8. 10.1182/blood-2010-10-311894 21292771

[8] Desreumaux P Foussat A Allez M Beaugerie L Hébuterne X Bouhnik Y Safety and efficacy of antigen-specific regulatory T-cell therapy for patients with refractory Crohn’s disease. *Gastroenterology.* (2012) 143:1207–17.e2. 10.1053/j.gastro.2012.07.116 22885333

[9] Marek-Trzonkowska N Myśliwiec M Dobyszuk A Grabowska M Derkowska I Juścińska J Therapy of type 1 diabetes with CD4(+)CD25(high)CD127-regulatory T cells prolongs survival of pancreatic islets - results of one year follow-up. *Clin Immunol Orlando Fla.* (2014) 153:23–30. 10.1016/j.clim.2014.03.016 24704576

[10] Bluestone J Buckner J Fitch M Gitelman S Gupta S Hellerstein M Type 1 diabetes immunotherapy using polyclonal regulatory T cells. *Sci Transl Med.* (2015) 7:315ra189. 10.1126/scitranslmed.aad4134 26606968 PMC4729454

[11] Bottomley M Harden P Wood K Hester J Issa F. Dampened inflammatory signalling and myeloid-derived suppressor-like cell accumulation reduces circulating monocytic HLA-DR density and associates with malignancy risk in long-term renal transplant recipients. *Front Immunol.* (2022) 13:901273. 10.3389/fimmu.2022.901273 35844527 PMC9283730

[12] Tang Q Bluestone J. Regulatory T-cell therapy in transplantation: moving to the clinic. *Cold Spring Harb Perspect Med.* (2013) 3:a015552. 10.1101/cshperspect.a015552 24186492 PMC3808774

[13] Chandran S Tang Q Sarwal M Laszik Z Putnam A Lee K Polyclonal regulatory T cell therapy for control of inflammation in kidney transplants. *Am J Transplant Off J Am Soc Transplant Am Soc Transpl Surg.* (2017) 17:2945–54. 10.1111/ajt.14415 28675676 PMC5662482

[14] Mathew J H-Voss J LeFever A Konieczna I Stratton C He J A phase I clinical trial with ex vivo expanded recipient regulatory T cells in living donor kidney transplants. *Sci Rep.* (2018) 8:7428. 10.1038/s41598-018-25574-7 29743501 PMC5943280

[15] Sawitzki B Harden P Reinke P Moreau A Hutchinson J Game D Regulatory cell therapy in kidney transplantation (The ONE Study): a harmonised design and analysis of seven non-randomised, single-arm, phase 1/2A trials. *Lancet Lond Engl.* (2020) 395:1627–39. 10.1016/S0140-6736(20)30167-7 PMC7613154 32446407

[16] Harden P Game D Sawitzki B Van der Net J Hester J Bushell A Feasibility, long-term safety, and immune monitoring of regulatory T cell therapy in living donor kidney transplant recipients. *Am J Transplant.* (2021) 21:1603–11. 10.1111/ajt.16395 33171020 PMC7613119

[17] Brook M Hester J Petchey W Rombach I Dutton S Bottomley M Transplantation Without Overimmunosuppression (TWO) study protocol: a phase 2b randomised controlled single-centre trial of regulatory T cell therapy to facilitate immunosuppression reduction in living donor kidney transplant recipients. *BMJ Open.* (2022) 12:e061864. 10.1136/bmjopen-2022-061864 35428650 PMC9014059

[18] Dri D Praticò G Gaucci E Marianecci C Gramaglia D. Quality assessment of investigational medicinal products in COVID-19 clinical trials: one year of activity at the clinical trials office. *Pharmaceuticals.* (2021) 14:1321. 10.3390/ph14121321 34959722 PMC8709226

[19] Raffin C Vo L Bluestone J. Treg cell-based therapies: challenges and perspectives. *Nat Rev Immunol.* (2020) 20:158–72. 10.1038/s41577-019-0232-6 31811270 PMC7814338

[20] Brunstein C Miller J McKenna D Hippen K DeFor T Sumstad D Umbilical cord blood-derived T regulatory cells to prevent GVHD: kinetics, toxicity profile, and clinical effect. *Blood.* (2016) 127:1044–51. 10.1182/blood-2015-06-653667 26563133 PMC4768428

[21] Parmar S Liu X Najjar A Shah N Yang H Yvon E Ex vivo fucosylation of third-party human regulatory T cells enhances anti-graft-versus-host disease potency in vivo. *Blood.* (2015) 125:1502–6. 10.1182/blood-2014-10-603449 25428215 PMC4342362

[22] Parmar S Liu X Tung S Robinson S Rodriguez G Cooper L Third-party umbilical cord blood-derived regulatory T cells prevent xenogenic graft-versus-host disease. *Cytotherapy.* (2014) 16:90–100. 10.1016/j.jcyt.2013.07.009 24480547 PMC4124936

[23] Amini L Greig J Schmueck-Henneresse M Volk H Bézie S Reinke P Super-treg: toward a new era of adoptive treg therapy enabled by genetic modifications. *Front Immunol.* (2021) 11:611638. 10.3389/fimmu.2020.611638 33717052 PMC7945682

[24] McCallion O Bilici M Hester J Issa F. Regulatory T-cell therapy approaches. *Clin Exp Immunol.* (2022) [Online ahead of print]. 10.1093/cei/uxac078 35960852 PMC10019137

[25] Mu J Tai X Iyer S Weissman J Singer A Singer D. Regulation of MHC class I expression by Foxp3 and its effect on Treg cell function. *J Immunol Baltim Md 1950.* (2014) 192:2892–903. 10.4049/jimmunol.1302847 24523508 PMC3952169

[26] Poirot L Jahangiri B Duchateau P Valton J. Allogeneic CAR T-cells resistant to both T- and NK-cell cytotoxicity. *Cytotherapy.* (2020) 22:S134–5. 10.1016/j.jcyt.2020.03.264

[27] Wang D Quan Y Yan Q Morales J Wetsel R. Targeted disruption of the β2-microglobulin gene minimizes the immunogenicity of human embryonic stem cells. *Stem Cells Transl Med.* (2015) 4:1234–45. 10.5966/sctm.2015-0049 26285657 PMC4572902

[28] Jaiswal S Jamieson C Pang W Park C Chao M Majeti R CD47 is upregulated on circulating hematopoietic stem cells and leukemia cells to avoid phagocytosis. *Cell.* (2009) 138:271–85. 10.1016/j.cell.2009.05.046 19632178 PMC2775564

[29] Gornalusse G Hirata R Funk S Riolobos L Lopes V Manske G HLA-E-expressing pluripotent stem cells escape allogeneic responses and lysis by NK cells. *Nat Biotechnol.* (2017) 35:765–72. 10.1038/nbt.3860 28504668 PMC5548598

[30] Deuse T Hu X Gravina A Wang D Tediashvili G De C Hypoimmunogenic derivatives of induced pluripotent stem cells evade immune rejection in fully immunocompetent allogeneic recipients. *Nat Biotechnol.* (2019) 37:252–8. 10.1038/s41587-019-0016-3 30778232 PMC6419516

[31] O’Neil A Brook M Abdul-Wahab S Hester J Lombardi G Issa F. A GMP protocol for the manufacture of tregs for clinical application. In: Ono M editor. *Regulatory T-Cells: Methods and Protocols. Methods in Molecular Biology.* New York, NY: Springer US (2023). p. 205–27. 10.1007/978-1-0716-2647-4_14 36180635

[32] Martelli M Di Ianni M Ruggeri L Falzetti F Carotti A Terenzi A HLA-haploidentical transplantation with regulatory and conventional T-cell adoptive immunotherapy prevents acute leukemia relapse. *Blood.* (2014) 124:638–44. 10.1182/blood-2014-03-564401 24923299

[33] Trzonkowski P Bacchetta R Battaglia M Berglund D Bohnenkamp H ten Brinke A Hurdles in therapy with regulatory T cells. *Sci Transl Med.* (2015) 7:304s18. 10.1126/scitranslmed.aaa7721 26355029

[34] Berglund D Karlsson M Biglarnia A Lorant T Tufveson G Korsgren O Obtaining regulatory T cells from uraemic patients awaiting kidney transplantation for use in clinical trials. *Clin Exp Immunol.* (2013) 173:310–22. 10.1111/cei.12112 23607776 PMC3722931

[35] Baecher-Allan C Wolf E Hafler D. Functional analysis of highly defined, FACS-isolated populations of human regulatory CD4+ CD25+ T cells. *Clin Immunol Orlando Fla.* (2005) 115:10–8. 10.1016/j.clim.2005.02.018 15870015

[36] Cossarizza A Chang H Radbruch A Acs A Adam D Adam-Klages S Guidelines for the use of flow cytometry and cell sorting in immunological studies (second edition). *Eur J Immunol.* (2019) 49:1457–973. 10.1002/eji.201970107 31633216 PMC7350392

[37] Gee A Durett A. Cell sorting for therapeutic applications - points to consider. *Cytotherapy.* (2002) 4:91–2. 10.1080/146532402317251608 11953049

[38] Jayasinghe S Wunderlich J McKee A Newkirk H Pope S Zhang J Sterile and disposable fluidic subsystem suitable for clinical high speed fluorescence-activated cell sorting. *Cytometry B Clin Cytom.* (2006) 70B:344–54. 10.1002/cyto.b.20111 16739216

[39] Keane-Moore M Coder D Marti G. Public Meeting and Workshop on “Safety issues pertaining to the clinical application of flow cytometry to human-derived cells.”. *Cytotherapy.* (2002) 4:89–90. 10.1080/146532402317251590 11953048

[40] Hickerson D Fiordalisi M Reese M Deibert E Balber A Kurtzberg J Modification of a commercial cell sorter to support efficient and reliable preparation of ALDH-bright cells for clinical use. *Cytotherapy.* (2007) 9:562–8. 10.1080/14653240701466321 17882721

[41] Canavan J Scottà C Vossenkämper A Goldberg R Elder M Shoval I Developing in vitro expanded CD45RA+ regulatory T cells as an adoptive cell therapy for Crohn’s disease. *Gut.* (2016) 65:584–94. 10.1136/gutjnl-2014-306919 25715355 PMC4819603

[42] Proics E David M Mojibian M Speck M Lounnas-Mourey N Govehovitch A Preclinical assessment of antigen-specific chimeric antigen receptor regulatory T cells for use in solid organ transplantation. *Gene Ther.* (2022):[Online ahead of print]. 10.1038/s41434-022-00358-x 35931871 PMC10113151

[43] Hulspas R Villa-Komaroff L Koksal E Etienne K Rogers P Tuttle M Purification of regulatory T cells with the use of a fully enclosed high-speed microfluidic system. *Cytotherapy.* (2014) 16:1384–9. 10.1016/j.jcyt.2014.05.016 25065635

[44] Duggleby R Danby R Madrigal J Saudemont A. Clinical grade regulatory CD4+ T Cells (Tregs): moving toward cellular-based immunomodulatory therapies. *Front Immunol.* (2018) 9:252. 10.3389/fimmu.2018.00252 29487602 PMC5816789

[45] Peters J Preijers F Woestenenk R Hilbrands L Koenen H Joosten I. Clinical grade treg: GMP isolation, improvement of purity by CD127pos depletion, treg expansion, and treg cryopreservation. *PLoS One.* (2008) 3:e3161. 10.1371/journal.pone.0003161 18776930 PMC2522271

[46] Elkord E. Frequency of human T regulatory cells in peripheral blood is significantly reduced by cryopreservation. *J Immunol Methods.* (2009) 347:87–90. 10.1016/j.jim.2009.06.001 19538964

[47] Sattui S de la Flor C Sanchez C Lewis D Lopez G Rizo-Patrón E Cryopreservation modulates the detection of regulatory T cell markers. *Cytometry B Clin Cytom.* (2012) 82B:54–8. 10.1002/cyto.b.20621 21936048

[48] Van Hemelen D Oude Elberink J Heimweg J van Oosterhout A Nawijn M. Cryopreservation does not alter the frequency of regulatory T cells in peripheral blood mononuclear cells. *J Immunol Methods.* (2010) 353:138–40. 10.1016/j.jim.2009.11.012 19919839

[49] Venet F Malcus C Ferry T Poitevin F Monneret G. Percentage of regulatory T cells CD4+CD25+CD127- in HIV-infected patients is not reduced after cryopreservation. *J Immunol Methods.* (2010) 357:55–8. 10.1016/j.jim.2010.02.005 20188734

[50] Kaiser D Otto N McCallion O Hoffmann H Zarrinrad G Stein M Freezing medium containing 5% DMSO enhances the cell viability and recovery rate after cryopreservation of regulatory T cell products ex vivo and in vivo. *Front Cell Dev Biol.* (2021) 9:750286. 10.3389/fcell.2021.750286 34926446 PMC8677839

[51] Gołąb K Grose R Placencia V Wickrema A Solomina J Tibudan M Cell banking for regulatory T cell-based therapy: strategies to overcome the impact of cryopreservation on the Treg viability and phenotype. *Oncotarget.* (2018) 9:9728–40. 10.18632/oncotarget.23887 29515766 PMC5839397

[52] Fraser H Safinia N Grageda N Thirkell S Lowe K Fry L A rapamycin-based GMP-compatible process for the isolation and expansion of regulatory T cells for clinical trials. *Mol Ther Methods Clin Dev.* (2018) 8:198–209. 10.1016/j.omtm.2018.01.006 29552576 PMC5850906

[53] Boardman D Philippeos C Fruhwirth G Ibrahim MA Hannen R Cooper D Expression of a chimeric antigen receptor specific for donor HLA Class I enhances the potency of human regulatory T cells in preventing human skin transplant rejection. *Am J Transplant Off J Am Soc Transplant Am Soc Transpl Surg.* (2017) 17:931–43. 10.1111/ajt.14185 28027623

[54] Huter E Stummvoll G DiPaolo R Glass D Shevach E. Cutting edge: antigen-specific TGF beta-induced regulatory T cells suppress Th17-mediated autoimmune disease. *J Immunol Baltim Md 1950.* (2008) 181:8209–13. 10.4049/jimmunol.181.12.8209 19050237 PMC2788513

[55] Tran G Hodgkinson S Carter N Verma N Robinson C Plain K Autoantigen specific IL-2 activated CD4+CD25+T regulatory cells inhibit induction of experimental autoimmune neuritis. *J Neuroimmunol.* (2020) 341:577186. 10.1016/j.jneuroim.2020.577186 32058174

[56] Stephens L Malpass K Anderton S. Curing CNS autoimmune disease with myelin-reactive Foxp3+ Treg. *Eur J Immunol.* (2009) 39:1108–17. 10.1002/eji.200839073 19350586

[57] Tang Q Henriksen K Bi M Finger E Szot G Ye J In vitro-expanded antigen-specific regulatory T cells suppress autoimmune diabetes. *J Exp Med.* (2004) 199:1455–65. 10.1084/jem.20040139 15184499 PMC2211775

[58] MacDonald K Hoeppli R Huang Q Gillies J Luciani D Orban P Alloantigen-specific regulatory T cells generated with a chimeric antigen receptor. *J Clin Invest.* (2016) 126:1413–24. 10.1172/JCI82771 26999600 PMC4811124

[59] Dawson N Lamarche C Hoeppli R Bergqvist P Fung V McIver E Systematic testing and specificity mapping of alloantigen-specific chimeric antigen receptors in regulatory T cells. *JCI Insight.* (2019) 4:e123672. 10.1172/jci.insight.123672 30753169 PMC6483008

[60] Jenkins M Moon J. The role of naive T cell precursor frequency and recruitment in dictating immune response magnitude. *J Immunol Baltim Md 1950.* (2012) 188:4135–40. 10.4049/jimmunol.1102661 22517866 PMC3334329

[61] Gaublomme J Yosef N Lee Y Gertner R Yang L Wu C Single-cell genomics unveils critical regulators of Th17 cell pathogenicity. *Cell.* (2015) 163:1400. 10.1016/j.cell.2015.11.009 26607794 PMC4671824

[62] Wang C Yosef N Gaublomme J Wu C Lee Y Clish C CD5L/AIM regulates lipid biosythesis and restrains Th17 cell pathogenicity. *Cell.* (2015) 163:1413. 10.1016/j.cell.2015.10.068 26607793 PMC4671820

[63] DuPage M Bluestone J. Harnessing the plasticity of CD4+ T cells to treat immune-mediated disease. *Nat Rev Immunol.* (2016) 16:149–63. 10.1038/nri.2015.18 26875830

[64] Balashov K Rottman J Weiner H Hancock W. CCR5+ and CXCR3+ T cells are increased in multiple sclerosis and their ligands MIP-1α and IP-10 are expressed in demyelinating brain lesions. *Proc Natl Acad Sci USA.* (1999) 96:6873. 10.1073/pnas.96.12.6873 10359806 PMC22009

[65] Christen U McGavern D Luster A von Herrath M Oldstone M. Among CXCR3 Chemokines, IFN-γ-Inducible Protein of 10 kDa (CXC Chemokine Ligand (CXCL) 10) but Not Monokine Induced by IFN-γ (CXCL9) Imprints a Pattern for the Subsequent Development of Autoimmune Disease. *J Immunol.* (2003) 171:6838–45. 10.4049/jimmunol.171.12.6838 14662890

[66] Guo J Zhou X. Regulatory T cells turn pathogenic. *Cell Mol Immunol.* (2015) 12:525–32. 10.1038/cmi.2015.12 25942597 PMC4579652

[67] Iglesias-Lopez C Agustí A Obach M Vallano A. Regulatory framework for advanced therapy medicinal products in Europe and United States. *Front Pharmacol.* (2019) 10:921. 10.3389/fphar.2019.00921 31543814 PMC6728416

[68] European Medicines Agency [EMA]. *Orphan designation: Overview.* (2020). Available online at: https://www.ema.europa.eu/en/human-regulatory/overview/orphan-designation-overview (accessed January 1, 2021).

[69] Food and Drug Administration [FDA]. *Rules and Regulations Federal Register Internet.* Silver Spring, MA: Food and Drug Administration (2013).

[70] Detela G Lodge A. EU Regulatory Pathways for ATMPs: standard, accelerated and adaptive pathways to marketing authorisation. *Mol Ther Methods Clin Dev.* (2019) 13:205–32. 10.1016/j.omtm.2019.01.010 30815512 PMC6378853

[71] European Medicines Agency [EMA]. *Advanced Therapies: Marketing Authorisation.* (2021). Available online at: https://www.ema.europa.eu/en/human-regulatory/marketing-authorisation/advanced-therapies/marketing-authorisation-procedures-advanced-therapy-medicinal-products (accessed January 1, 2021).

[72] Ten Ham R Hoekman J Hövels A Broekmans A Leufkens H Klungel O. Challenges in advanced therapy medicinal product development: a survey among companies in Europe. *Mol Ther Methods Clin Dev.* (2018) 11:121–30. 10.1016/j.omtm.2018.10.003 30456217 PMC6234262

[73] Eu Monitor. *Regulation 2020/1043 - Conduct of Clinical Trials with and Supply of Medicinal Products for Human use Containing or Consisting of Genetically Modified Organisms Intended to Treat or Prevent Coronavirus Disease (COVID-19) - EU monitor.* (2022). Available online at: https://www.eumonitor.eu/9353000/1/j9vvik7m1c3gyxp/vlafbrflekzq (accessed October 2, 2022).

[74] Beattie S Hubert A Morrell J Cook J Werner M Ginty P Call for more effective regulation of clinical trials with advanced therapy medicinal products consisting of or containing genetically modified organisms in the European Union. *Hum Gene Ther.* (2021) 32:997–1003. 10.1089/hum.2021.058 33843251

[75] Pizevska M Kaeda J Fritsche E Elazaly H Reinke P Amini L. Advanced therapy medicinal products’ translation in Europe: a developers’ perspective. *Front Med.* (2022) 9:757647. 10.3389/fmed.2022.757647 35186986 PMC8851388

[76] Regier D Pollard S McPhail M Bubela T Hanna T Ho C A perspective on life-cycle health technology assessment and real-world evidence for precision oncology in Canada. *NPJ Precis Oncol.* (2022) 6:76. 10.1038/s41698-022-00316-1 36284134 PMC9596463

[77] Facey K Rannanheimo P Batchelor L Borchardt M de Cock J. Real-world evidence to support Payer/HTA decisions about highly innovative technologies in the EU-actions for stakeholders. *Int J Technol Assess Health Care.* (2020) [Online ahead of print]. 10.1017/S026646232000063X 32878663

[78] Bell R Roberts C. UK at the forefront of advanced therapies. *Eur Pharm Rev.* (2021) 26:56–60.

[79] Burki TK. UK to align with EU clinical trial rules post-Brexit. *Lancet Oncol.* (2018) 19:e289. 10.1016/S1470-2045(18)30327-9 29706378

[80] European Economic Community. *Commission Directive 91/356/EEC of 13 June 1991 Laying Down the Principles and Guidelines of Good Manufacturing Practice for Medicinal Products for Human Use.* (1991). Available online at: https://op.europa.eu/en/publication-detail/-/publication/dd7e01a5-6dba-4520-b573-0cd82b39d5dd/language-en (accessed June 13, 1991).

[81] Codinach M Blanco M Ortega I Lloret M Reales L Coca M Design and validation of a consistent and reproducible manufacture process for the production of clinical-grade bone marrow-derived multipotent mesenchymal stromal cells. *Cytotherapy.* (2016) 18:1197–208. 10.1016/j.jcyt.2016.05.012 27424149

[82] de Wilde S Veltrop-Duits L Hoozemans-Strik M Ras T Blom-Veenman J Guchelaar H Hurdles in clinical implementation of academic advanced therapy medicinal products: a national evaluation. *Cytotherapy.* (2016) 18:797–805. 10.1016/j.jcyt.2016.02.010 27068764

[83] Van Wilder P. Advanced therapy medicinal products and exemptions to the regulation 1394/2007: how confident can we be? An exploratory analysis. *Front Pharmacol.* (2012) 3:12. 10.3389/fphar.2012.00012 22347860 PMC3278850

[84] Tsiftsoglou A Ruiz S Schneider C. Development and regulation of biosimilars: current status and future challenges. *BioDrugs Clin Immunother Biopharm Gene Ther.* (2013) 27:203–11. 10.1007/s40259-013-0020-y 23553340

[85] European Commission. *Final Report Summary - AGORA (ATMP GMP Open Access Research Alliance - AGORA) | FP7 | CORDIS |.* (2022). Available online at: https://cordis.europa.eu/project/id/602366/reporting (accessed October 2, 2022).

[86] Evaluate Company. *Evaluate Pharma World Preview 2020, Outlook to 2026.* (2020). Available online at: https://www.evaluate.com/thought-leadership/pharma/evaluatepharma-world-preview-2020-outlook-2026 (accessed October 9, 2022).

[87] McKinsey Company. *Opportunities and challenges for Cell Gene Therapies in Pharmaceuticals and Medical Products in Europe.* (2021). Available online at: https://www.mckinsey.com/industries/life-sciences/our-insights/a-call-to-action-opportunities-and-challenges-for-cgts-in-europe (accessed August 19, 2022).

[88] Fink J Scott M Rieck S Jones R Parisse J Hagen H Impact considerations of post-production processes on cell and gene drug products. *Cytotherapy.* (2022) 24:583–9. 10.1016/j.jcyt.2021.12.012 35643522

[89] McKinsey. *C19 and Cell and Gene Therapy.* (2020). Available online at: https://www.mckinsey.com/industries/life-sciences/our-insights/covid-19-and-cell-and-gene-therapy-how-to-keep-innovation-on-track (accessed October 9, 2022).

[90] Coleman Parks. *Tomorrows Supply Chain: Disruption around every Corner.* (2022). Available online at: https://www.sap.com/uk/documents/2022/06/bc551cb1-2e7e-0010-bca6-c68f7e60039b.html (accessed January 11, 2022).

[91] European Medicines Agency. *CAT-Quarterly-Highlights-Approved-Atmps.* (2022). Available online at: https://www.ema.europa.eu/en/documents/report/cat-quarterly-highlights-approved-atmps-july-2022_en.pdf (accessed October 9, 2022).

[92] Champion A Lewis S Davies S Hughes D. Managing access to advanced therapy medicinal products: challenges for NHS Wales. *Br J Clin Pharmacol.* (2021) 87:2444–9. 10.1111/bcp.14286 32495405

[93] McCabe C Claxton K Culyer A. The NICE cost-effectiveness threshold: what it is and what that means. *PharmacoEconomics.* (2008) 26:733–44. 10.2165/00019053-200826090-00004 18767894

[94] Wood E Hughes D. The new and non-transparent cancer drugs fund. *PharmacoEconomics.* (2020) 38:1–4. 10.1007/s40273-019-00871-9 31828737

[95] National Institute for Health and Clinical Excellence. *Interim Process and Methods of the Highly Specialised Technologies Programme Updated to reflect 2017 Changes.* London: National institute for health and clinical excellence (2017). 27905709

[96] Hatswell A Freemantle N Baio G. Economic evaluations of pharmaceuticals granted a marketing authorisation without the results of randomised trials: a systematic review and taxonomy. *PharmacoEconomics.* (2017) 35:163–76. 10.1007/s40273-016-0460-6 27778240

[97] Jørgensen J Kefalas P. Annuity payments can increase patient access to innovative cell and gene therapies under England’s net budget impact test. *J Mark Access Health Policy.* (2017) 5:1355203. 10.1080/20016689.2017.1355203 28839525 PMC5560408

[98] Jørgensen J Hanna E Kefalas P. Outcomes-based reimbursement for gene therapies in practice: the experience of recently launched CAR-T cell therapies in major European countries. *J Mark Access Health Policy.* (2020) 8:1715536. 10.1080/20016689.2020.1715536 32082514 PMC7006635

[99] Jørgensen J Kefalas P. Upgrading the SACT dataset and EBMT registry to enable outcomes-based reimbursement in oncology in England: a gap analysis and top-level cost estimate. *J Mark Access Health Policy.* (2019) 7:1635842. 10.1080/20016689.2019.1635842 31303982 PMC6609347

[100] Apm Health Europe. *Major German Payers Sign Pay for Performance Agreements on CAR-Ts.* (2022). Available online at: https://www.apmhealtheurope.com/freestory/0/64434/major-german-payers-sign-pay-for-performance-agreements-on-car-ts (accessed October 9, 2022).

[101] Novartis. *Novartis 2017 US Transparency and Patient Access Report.* Bengaluru: Novartis India Limited (2017).

[102] Centers for Medicare & Medicaid Services [CMS]. *Proposed Decision Memo for Chimeric Antigen Receptor (CAR) T-cell Therapy for Cancers.* Baltimore, MD: Centers for Medicare & Medicaid Services (2019).

[103] Gold E. Accelerating translational research through open science: the neuro experiment. *PLoS Biol.* (2016) 14:e2001259. 10.1371/journal.pbio.2001259 27932848 PMC5147793

[104] Gold E. The fall of the innovation empire and its possible rise through open science. *Res Policy.* (2021) 50:104226. 10.1016/j.respol.2021.104226 34083844 PMC8024784

